# Liver macrophages: development, dynamics, and functions

**DOI:** 10.1038/s41423-025-01298-3

**Published:** 2025-06-03

**Authors:** Ysbrand Nusse, Paul Kubes

**Affiliations:** 1 Calvin, Phoebe, and Joan Snyder Institute for Chronic Diseases, Cumming School of Medicine, University of Calgary, Calgary, AB Canada; 2 Department of Physiology and Pharmacology, Cumming School of Medicine, University of Calgary, Calgary, AB Canada; 3 Department of Biomedical and Molecular Science, Queen’s University, Kingston, ON Canada

**Keywords:** Macrophage, Liver, Kupffer cell, Monocyte-derived macrophage, Liver fibrosis, Kupffer cells, Imaging the immune system

## Abstract

The liver is a sizeable visceral organ whose primary functions involve nutrient metabolism, clearance of toxins, and energy storage. Besides these critical functions, the liver is also a major immunological site. It is populated by several specialized resident immune cells, including B cells, T Cells, dendritic cells, and several populations of macrophages. It is also the site for the production and release of acute-phase proteins during inflammation. One reason for garrisoning these immune sentinels and effectors in the liver is its relative location in the circulatory system. The liver is the first significant organ downstream of the intestine, where blood originating from the intestine enters the liver through the portal vein. This organization facilitates the liver’s uptake and processing of nutrient-rich blood directly from the intestinal source. However, the intestine is also home to trillions of microbes, many of which are commensals but also represent potential pathogens. As such, the portal blood supply represents an avenue for systemic infection. To sterilize the portal blood, the liver immune system filters pathogens, which is primarily accomplished by liver macrophages. Here, we will discuss the major populations of macrophages resident in the liver, their location, functions, development, and role in maintaining the liver in the face of injury and infection.

## Introduction: Liver structure and cell types

Liver macrophages do not exist in a vacuum. Like other cells, immune or otherwise, they depend on and contribute to their local microenvironment. Signals from non-immune cells are critical for the development and function of liver macrophages, and likewise, liver macrophages imprint upon and shape the behavior of their neighboring cells. As such, we will briefly describe the non-immunological components of the liver, which are essential for supporting liver immunity.

**Fig. 1:**
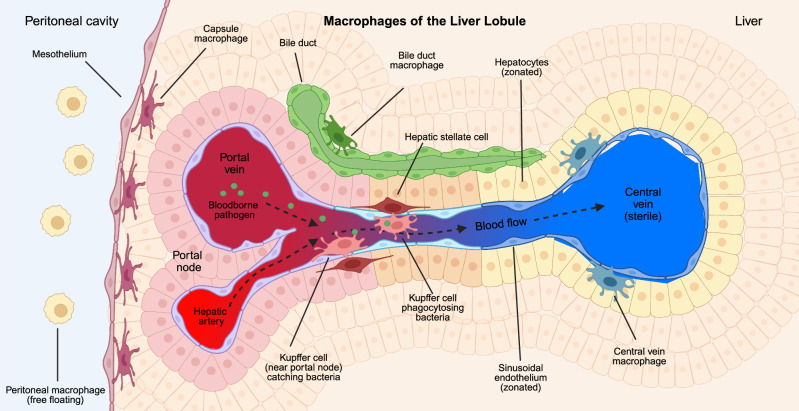
Macrophages of the liver. At least 4 types of liver macrophages reside in the liver. Kupffer cells exist in the sinusoids, near the portal node, where they can catch and phagocytose bloodborne pathogens. Liver capsule macrophages reside beneath the mesothelium on the liver surface. Central vein macrophages are found near the central vein, while bile duct macrophages cluster near epithelial bile ducts. Mixed blood flows from the portal vein and hepatic artery towards the central vein, passing by zonated endothelial cells and hepatocytes. Outside of the liver, peritoneal macrophages are free floating within the peritoneal cavity. Created in BioRender. Nusse, Y. (2025) https://BioRender.com/fpzrxhl

The liver’s structure elegantly enables the multiple functions of the organ. A unique vasculature system infiltrates the liver, featuring a mixed blood supply that draws venous flow from the intestine through the portal vein, which is mixed with oxygenated blood from the hepatic artery at a structure termed the portal node [1] (Fig. 1). There, nutrient-rich mixed blood passes through a network of specialized capillaries called sinusoids before draining into the central vein. Between the portal and central vein, the liver is organized into repeating functional units within the liver - the liver lobules [2]. Liver lobules are canonically considered repetitive hexagonal units organized around the central vein. The sinusoidal endothelium between the portal node and central vein is lined with parenchymal cells carrying out the liver’s critical functions. These parenchymal cells are *zonated*, meaning their functions and gene expression are polarized between the central vein and portal node [3, 4].

The primary cells lining the sinusoids are hepatocytes. Hepatocytes are significant sites of metabolic activity, energy storage, and clearance of toxins. The zonation of hepatocytes enables them to serve different functions depending on their location between the portal and central vein [4, 5]. The gene signatures for portal hepatocytes are enriched for transcripts associated with gluconeogenesis, cholesterol synthesis, and metabolism of fatty acids and amino acids [6–9]. Hepatocytes near the central vein are enriched for the expression of genes related to glycolysis, bile acid production, and detoxification. Meanwhile, hepatocytes in the middle layers participate in iron homeostasis and insulin. The zonation of hepatocytes facilitates the liver’s function in nutrient metabolism. The relationship and organization of hepatocyte zonation is an area of active ongoing exploration facilitated by recent advances in spatially resolved RNAseq datasets [10–12]. Hepatocytes are also famously regenerative and can enter the cell cycle or undergo cell hypertrophy to restore the liver after significant damage [13–23].

The liver sinusoidal endothelial cells (LSECs) are also zonated. Periportal, mid-lobular, and pericentral LSECs express distinct markers. Periportal LSECs are enriched for genes such as *Ltbp4*, *Efnb2*, and *Dll4* [24]. *Dll4* is especially interesting to consider as a zonated LSEC gene because, as discussed below, *Dll4* is a key component of the macrophage niche in the liver [25], implying that the macrophage niche is itself zonated. Notably, the pericentral endothelium is a key source of essential growth signals, such as Wnt ligands, which are thought to drive the zonation of hepatocytes [16, 24, 26–28].

Besides the hepatocytes, other parenchymal cell populations within the liver include hepatic stellate cells, the fibroblasts of the liver, and the biliary epithelium. Hepatic stellate cells are important secretors of growth factors for hepatocytes and other liver cell types. They help control blood flow due to their contractile capabilities and serve as reservoirs of retinol [29–32]. Hepatic stellate cells are also important cells in liver repair and regeneration. Upon injury, hepatic stellate cells become activated and differentiate into highly contractile myofibroblasts implicated in acute injury repair and can contribute to liver scarring and fibrosis [29, 31, 33, 34]. Recently, hepatic stellate cells were shown to be essential drivers of liver zonation through the secretion of modulators of the Wnt signaling pathway [35].

The biliary epithelium, or cholangiocytes, are ductal cells that secrete and transport bile in the reverse direction of blood flow, providing key components aiding digestion [36]. These epithelial cells are considered quite plastic and may be able to differentiate into diverse cell types to promote liver repair, especially in chronic injury [37–39].

Outside the network of liver lobules, the liver is covered by a capsule, which is composed of a single-cell layered epithelium of mesothelial cells overlaying a fibrous layer containing fibroblasts [40]. Mesothelial cells provide a barrier and lubricating functions between other visceral organs. During injuries, especially intra-abdominal surgery, mesothelial cells undergo an epithelial-to-mesenchymal transition and can contribute to pathological scarring and adhesion formation [41, 42].

Beyond these hallmark liver cells, the liver is innervated with neuronal cells, which participate in metabolic homeostasis and may assist with injury repair [43–46]. Additionally, the liver is home to many types of immune cells beyond macrophages [47, 48]. Liver myeloid cells include dendritic cells [49] and MDSCs [50], while several lymphoid populations are resident as well, including Natural Killer Cells [51], NK T cells [52] and invariant NK T Cells [53, 54], CD4 and CD8 T-cells [55], and a large population of B cells [56].

Overall, liver parenchymal cells cooperate to enable the liver’s metabolic functions. However, they are also neighbors to several populations of macrophages, which, in addition to their classic roles in immunity, support the liver’s functions.

## Liver macrophages: markers, location, and homeostatic functions

The liver is not home to just one type of macrophage. Specific subtypes of liver macrophages can be distinguished from within this pool based on their location, markers, and functions. Classically, macrophages across organs express markers such as F4/80, CD64, MerTK, and Iba1 [57–61]. Liver macrophages also express these markers, but several distinct populations can be discerned within this population (Table 1).

**Table 1:** Markers and Location of Macrophages in the liver

	General Macrophage	Kupffer Cells	Capsule Macrophage	Central Vein Macrophage	Bile Duct Macrophage
**Location**	Located Systemically	Sinusoidal lumen, zonated portally	Liver capsule	Central vein	Bile ducts
**Markers**	F4/80 CD64 MerTK IBA1	Tim4 Clec4f Clec2 CRIg/*VSIG4* *FOLR2* *CD163* *CD5L* *MARCO*	CX3CR1 CD11c	CX3CR1	*Trem2* *Spp1* *Gpnmb1* *CD9*

Locations of liver macrophages, and the genes and proteins that distinguish them

### Kupffer cells

The macrophages most notably associated with the liver are the Kupffer cells [62]. Kupffer cells can be distinguished from other liver macrophages based on their location and expression of the surface molecules Tim4, CRIg/*Vsig4*, Clec2, and Clec4f in mice [25, 63]. In addition, recent single-cell studies have established that Kupffer cells express a core set of marker genes controlled by key transcription factors specific to the mature Kupffer cell state. These include the marker genes *CD5L, VSIG4, MARCO, CD163, and FOLR2* in both humans and mice [25, 63] (Table 1*)*. It is important to note that the expression of marker genes (and their detectability, depending on the methodology used) and their corresponding protein products may be discordant, and lead to divergent interpretations of cell classification.

Kupffer cells reside within the liver lobules and are positioned strategically within the lumen of the sinusoidal endothelium (Fig. 1). Kupffer cells adapt a stelliform shape within the sinusoids with several projecting pseudopods. Kupffer cells are thought to be long-lived cells that can self-replicate and are largely immotile. The precise location of Kupffer cells vis-à-vis the sinusoids has been a matter of some confusion. Initially, cells thought to be Kupffer cells were observed crawling along the sinusoidal endothelium [64] while others contended Kupffer cells were stationary [65]. Intravital microscopy confirmed that Kupffer cells are largely immotile and sit within the vasculature [66]. Recently, it has come to light that while the Kupffer cell body and nucleus primarily sit inside of the blood flow and adhere to the sinusoidal endothelium, their pseudopods can project outside of the endothelium to contact many of the liver parenchymal cells, including the hepatic stellate cells within the space of Disse, and hepatocytes [67]. This delicate arrangement facilitates the various functions of Kupffer cells, their maintenance through contact with their stromal niche, and provides avenues for crosstalk between the Kupffer cell and their non-immune neighbors (discussed below).

A key function of liver Kupffer cells is to sterilize blood by phagocytosing circulating pathogens and foreign particles. As alluded to above, portal blood draining through the liver from the intestine is potentially a significant avenue for pathogen dissemination. Since Kupffer cells are strategically located throughout the liver sinusoids, their station effectively forces bloodborne pathogens to pass by these highly effective immune sentinels. Upon a bloodstream infection, a large percentage of circulating bacteria are captured by Kupffer cells within the first pass through the liver [66, 68, 69]. This function is essential, as mice depleted for their Kupffer cells exhibit 100% mortality within 48 h of a bloodstream *Staphylococcus aureus* infection due to dissemination to other organs [70]. Kupffer cells are armed with several key molecules to recognize, bind, phagocytose, and eventually eliminate pathogens. Complement Receptor of Immunoglobulin superfamily (CRIg), one of the distinguishing markers of Kupffer cells, is a receptor that can bind to bacterial pathogens such as *Staph aureus* [69]. CRIg is a complement receptor with biophysical properties of a catch-bond, meaning it can catch under high flow dynamic conditions. Its capacity for binding bacteria depends not only on complement [71] but potentially other molecules including lipoteichoic acid, a cell wall component in Gram-positive bacteria [69]. Kupffer cells also express numerous scavenger receptors, and while their targets are unclear, they could be important for viral or fungal clearance [72, 73]. Kupffer cells in female mice also have a receptor for natural antibodies (IgM, IgG3), which confers a natural protection to females and their offspring [74]. Kupffer cells also express a number of pattern recognition receptors that recognize bacteria pathogen associated molecular patterns such as Toll-like receptors and others [75–77]. Upon pathogen recognition, Kupffer cells can coordinate immune responses through cytokine release, and are potent secretors of TNFα, IL-1, IL-6, IL-10, IL-12, among others [78–82]. Additionally, Kupffer cells are armed with many effector molecules that can efficiently eradicate phagocytosed bacteria. Kupffer cells are ladened with specialized vesicles termed phagolysosomes that harbor lysosomal enzymes and reactive oxygen species (ROS) which can kill bacteria [68].

Beyond maintaining blood sterility, Kupffer cells also support homeostatic functions. Kupffer cells remove aged platelets from circulation [83, 84]. During cell aging, platelets become desialylated, a loss of terminal sialic acid moieties on glycoproteins on their membrane [85]. This leads to recognition of the aged platelets by macrophages in both the liver and spleen. Aged platelets rapidly attach to liver Kupffer cells, and are internalized [83]. Interference with this process, via Kupffer cell depletion or deletion of key Kupffer cell receptors such as Clec4f, the Ashwell-Morell receptor, and Macrophage galactose lectin, resulted in platelet accumulation and abnormal bleeding [83, 84]. Kupffer cells have also been shown to be a site for the clearance of aged red blood cells (RBCs), but this aspect is less clear [86, 87]. As there is little debate that splenic macrophages remove old RBCs from circulation, liver macrophages may function as either complementary or backup system that only engages under certain conditions such as splenectomy [88]. Accordingly, Kupffer cells express many factors involved in iron metabolism and processing [89].

Akin to their capacity in clearing aged RBCs and platelets, Kupffer cells also have critical anti-tumor functions, in both coordinating immune responses to tumors [90] and directly phagocytosing circulating tumor cells [91]. The latter ability is driven by key Kupffer cell identity transcription factors, which repress SIRPα, a receptor of the CD47 “don’t eat me” signal often expressed by cancers [92–94]. Thus, Kupffer cells recognize and phagocytose cancer cells despite their expression of CD47. In fact, driving a Kupffer cell-like state in other macrophages can endow these cells with many of the same anti-tumoral capabilities as Kupffer cells [91]. However, because of their inability to move, tumors cells that attach to sinusoids can grow near immobilized Kupffer cells. Coating tumor cells with antibody allows the Kupffer cells to detect and reach towards the tumor cells, an event that may be mediated by complement activation [95].

In recent years, advanced single-cell technologies have identified potential sub-types of cells previously thought to be identical. Several single-cell studies probing both human and mouse Kupffer cells have suggested that subtypes of Kupffer cells may exist [96–98]. These subsets are suggested to have distinct immunomodulatory effects, metabolic functions, antigen presentation capacity, or abilities to capture bacteria, and are marked by *Marco*, *CD36,* or *CD206*. However, whether these subtypes of Kupffer cells represent *bone-fide* separate cell states, or a transitory state remains unclear [25, 63, 99].

Like other cells in the liver, Kupffer cells themselves are also zonated, and are enriched in the sinusoids near the portal vein [11, 96, 100]. Kupffer cells’ regionalization depends on sensing microbial products in the portal blood by the sinusoidal endothelium [100, 101]. This circuit promotes host defense by optimally positioning Kupffer cells around the portal vein from which pathogens are likely to originate and establishes an immunosuppressive zone that limits inflammation in response to bacterial stimuli [96].

### Liver capsule macrophages (LCMs)

A second significant population of macrophages resides not within the sinusoids of the liver but rather is restricted to its surface, which interfaces with the peritoneal cavity and potentially other visceral organs. The liver capsule is a structure formed by epithelial mesothelial cells, fibroblasts, and other cells and defines the boundary of the organ. There, liver capsule macrophages extend pseudopods to form a network that extends across the surface of the liver (Fig. 1). Like other macrophages, LCMs express F4/80 and CD64 but lack the Kupffer cell markers CRIg and Tim4 [102, 103]. LCMs can be distinguished by their expression of CX3CR1, and low levels of CD11c [103] (Table 1).

Functionally, LCMs are a relatively unknown population of cells. They can capture bacteria from the abdominal cavity, and the selective loss of LCMs (through administration of an anti-CSF-1R antibody) promotes bacterial dissemination from the peritoneal cavity into the liver [102]. LCM pseudopods reach both the peritoneal cavity and the blood, suggesting they could be important means of communication between these two spaces. However, their role in supporting the non-immune functions of the liver in homeostasis and their capabilities in response to injury remains largely unexplored.

### Other liver macrophages

Kupffer cells and LCMs are the dominant populations of macrophages within the homeostatic liver, with Kupffer cells being the most frequent. However, other relatively small populations of liver resident macrophages have been identified recently, often by high-resolution single-cell applications characterizing the immune milieu in the liver. One such small set of macrophages has been identified clustered near the  central vein [25] (Fig. 1). These cells express similar markers as Liver Capsule Macrophages, such as CX3CR1 (Table 1). These central vein macrophages are clearly localized in a very different locale than LCMs but are difficult to discern from each other by their gene expression signatures [25]. These cells remain enigmatic in their function and precise identity compared with other liver macrophages.

Another small population of macrophages in the homeostatic liver appears to be located near the epithelial bile ducts, which also share many of the same markers as lipid-associated macrophages (LAMs), which have been described in other organs [25, 104–107] (Fig. 1). These interesting macrophages are identified by their expression of the markers *Gpnmb1*, *Spp1, Trem2*, and *CD9* (Table 1). As discussed below, liver LAMs appear to expand during various drivers of liver injury but are clearly also present in the homeostatic liver. As with the LCM-like central vein macrophages, liver bile duct LAMs remain poorly understood, especially during homeostasis.

## Development of liver macrophages

Historically, it was believed that all adult macrophages were constantly replaced by circulating monocytes derived from hematopoietic stem cells located in the bone marrow [108]. However, we now know that while some populations of tissue macrophages are continually refreshed from Ly6C^+^ CCR2^+^ monocytes, such as intestinal macrophages [109], other types of macrophages seed organs early in life and are maintained by other mechanisms, such as microglia [110]. Within the liver, several strategies for maintaining the distinct populations of macrophages are used, which we discuss below.

**Fig. 2:**
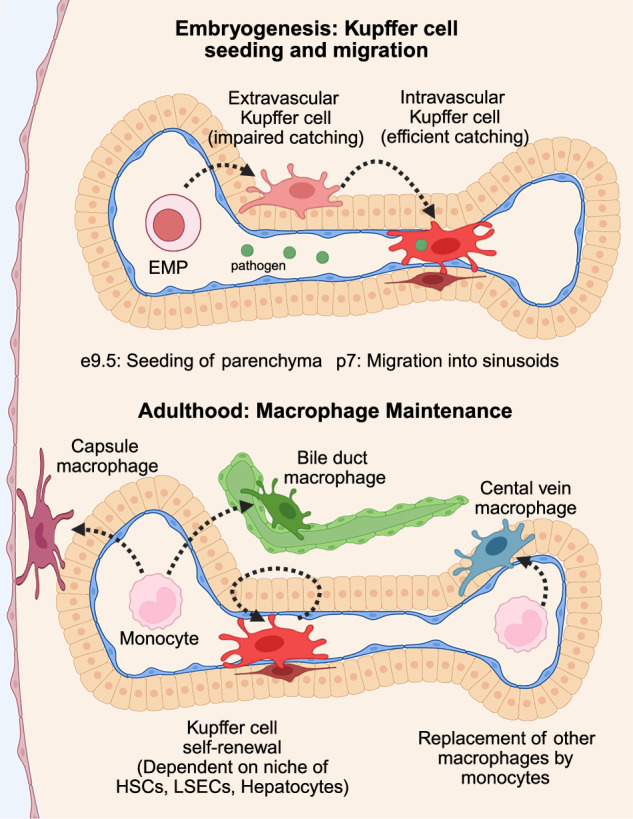
Development and maintenance of liver macrophages. During embryogenesis (top) erythro-myeloid progenitors (EMPs) seed the liver at around embryonic day 9.5 (e9.5) in mice. Developing Kupffer cells initially reside outside the vasculature, where they are unable to catch bacteria. At postnatal day 7 (p7) in mice, they migrate into the sinusoids, where they can catch bacteria. In adulthood (bottom), Kupffer cells are maintained through self-renewal, dependant on their niche, including hepatic stellate cells, liver sinusoidal endothelial cells (LSECs), and hepatocytes. Other populations of liver macrophages (capsule, central vein, and bile duct macrophages) are maintained by monocytes, which differentiate into macrophages. Created in BioRender. Nusse, Y. (2025) https://BioRender.com/ythmtwy

Kupffer cells are seeded in the liver early during development. In mice, *Tie2*^+^ *C-Myb*^+^ yolk sac hematopoietic erythro-myeloid progenitors (EMPs) seed the liver around embryonic day 8.5 and form primordial Kupffer cells [111–113] (Fig. 2). Upon seeding, EMPs begin differentiating towards a Kupffer cell specific state, featuring the several canonical transcription factors (such as *Id3*), extra-cellular receptors, and functional components discussed above [112, 113]. During development, Kupffer cells are distinct from another critical population of macrophages which support hematopoiesis which occurs in the fetal liver [114–116]. Recently, it was demonstrated that a key feature of Kupffer cell function, their ability to sterilize the bloodstream by catching blood-borne bacteria, is established somewhat late in their development [117]. By imaging the livers of newborn (postnatal day 1) mice, it was demonstrated that immature Kupffer cells do not efficiently capture blood-borne pathogens compared with adult mice despite being armed with many of the same molecules. Furthermore, newborn Kupffer cells had not fully elaborated their pseudopods or adopted a stelliform shape and were located mainly outside the sinusoidal endothelium (Fig. 2). By postnatal day 7, most Kupffer cells had migrated into the sinusoids and could more efficiently capture bacteria. The migration of these immature Kupffer cells into the sinusoids depended on several extracellular signals, including MIF signaling through CD74 and CD44. This study demonstrated that the precise location of the Kupffer cell is a critical factor in its ability to catch and eradicate pathogens. Additionally, as discussed above, Kupffer cells are zonated. This was suggested to occur by the recruitment of fetal derived macrophages to the proximal parts of sinusoids due to a chemokine gradient established by increased glycocalyx in this region [100]. Integrating these two concepts, whether young Kupffer cells crawl towards the portal region of the sinusoids after reverse transmigration, or are initially seeded near the portal node is not clear.

During adulthood, unlike macrophages from other organs, such as the intestine [118], liver Kupffer cells are relatively quiescent. They are not rapidly replaced by monocytes during homeostasis and rather can self-renew to refresh the pool of Kupffer cells over a long period [111, 119]. Lineage tracing studies using genetic labeling of monocytes have shown that while monocytes *can* replace Kupffer cells over the lifespan of mice, the percentage of Kupffer cells derived from monocytes is negligible and does not radically increase with age [120]. One open question is whether the slow rate of Kupffer cell turnover is an artifact of the relatively sterile environment that laboratory mice live in. The rate of Kupffer cell turnover in humans is presently not well understood. Studies in mice reconstituted with a wild microbiome and humans should shed light on whether Kupffer cells exposed to a higher pathogen load behave similarly to typical lab mice.

### The Kupffer cell niche

While Kupffer cells do not turn over rapidly during homeostasis, upon loss of the Kupffer cell population, a massive mobilization of monocytes occurs, rapidly replacing the absent macrophages. Within 24 h of macrophage depletion, monocytes are recruited to the liver, home to sites within the sinusoids, arrest, and begin differentiating into macrophages. This process is heavily regulated by the availability of a suitable niche [121]. Laboratory tools that allow the depletion of Kupffer cells, such as the administration of clodronate-loaded liposomes or a genetic strategy mediated by a diphtheria toxin receptor driven by the Kupffer cell-specific gene *Clec4f*, have facilitated the identification of niche factors that support the specification of Kupffer cells from monocytes [67, 122–124]. While this is an area of ongoing investigation, several studies have identified key signaling factors and cells that compose the Kupffer cell niche.

Upon Kupffer cell ablation in *Clec4f-DTR* mice, monocytes recruited to the Kupffer cell niche rapidly arrest, extend pseudopods, and adopt a Kupffer cell phenotype [67, 123]. The projections of Kupffer cells are especially important because they facilitate the physical interaction between the differentiating monocyte-derived Kupffer cell and its niche [67]. Interestingly, while several transcription factors governing the Kupffer cell state were rapidly induced in arrested monocytes, markers of Kupffer cell maturity took several days or weeks to establish fully. Bioinformatic tools have identified potential ligand-receptor interactions between the Kupffer cell and its niche. One such pairing occurs between the Kupffer cell and hepatic stellate cells, in which Kupffer cell pseudopods extend across the space of Disse to touch hepatic stellate cells [67]. This interaction may supply the developing Kupffer cell with CSF1, an important macrophage growth factor. Hepatic stellate cells also express several Bone Morphogenic Proteins (BMPs). Similarly, other nearby neighbors of the Kupffer cells, the LSECs, express Notch ligands, including DLL1 and DLL4, as well as BMPs and TGF-β [67, 123]. Functionally, culturing isolated monocytes with LSECs induced the expression of *Nr1h3 (*LXRα), *Rxra*, and *Spic*, hepatocyte co-culture induced expression of the key transcription factor *Id3* [67]. Induction of DLL4 in culture drove expression of *Nr1h3* and *Spic*, while blocking Notch ligands in vivo prevented the induction of these transcription factors in differentiating monocytes [123].

It is important to note that many of these studies specifically identify factors that facilitate the differentiation of monocytes into Kupffer cells. Whether these factors are also crucial in supporting yolk-sac-derived Kupffer cells is unclear. Furthermore, given the genetic strategy used to deplete Kupffer cells, it is difficult to prove that these factors act as part of the Kupffer cell niche in vivo because specifically deleting candidate factors using conditional knockouts becomes onerous when combined with the *Clec4f-DTR* strategy. Administration of clodronate-loaded liposomes or another drug-mediated ablation strategy may be viable but complicated by the non-specific nature of these approaches, which may have unwanted effects on monocytes as well.

Studies utilizing Kupffer cell depletion and reconstitution have identified several key ligands, receptors, and transcription factors specifying the Kupffer cell state, including the transcription factors *Spic*, *Nr1h3 (*LXRα), *Tcf7l2*, and *Id3* [67, 91, 117, 123, 125, 126]. In addition, several other key transcription factors are critical in macrophage biology, including PU.1 [127] and Zeb2 [128]. PU.1 and Zeb2 are key transcription factors specifying macrophage identity across tissues [128, 129]. In the liver, genetic deletion of *Zeb2* does not alter the total number of hepatic macrophages but does reduce their expression of the key Kupffer cell markers Clec4f and Tim4. Concurrently, *Zeb2* mutant Kupffer cells upregulated SiglecF and CD20, markers not typically associated with Kupffer cells. This also reduced their fitness, as *Zeb2* knockout macrophages were lost in the liver over time and were eventually replaced by other macrophages [128].

### Development and niche of other liver macrophages

Compared with Kupffer cells, the development, specification, and niche of other liver macrophages are relatively unexplored.

Unlike Kupffer cells derived from yolk sac macrophages seeded during embryogenesis and primarily maintained in homeostasis, liver capsule macrophages are continuously replaced by blood-derived monocytes and are mainly established after birth [102] (Fig. 2). The full establishment of the LCM pool occurs upon weaning and the acquisition of the microbiome, suggesting bacterial signals are essential regulators of the LCM pool [102]. Indeed, treatment with antibiotics reduces the frequency of LCMs [102]. The cells and signals that maintain LCMs are murky, although they are highly sensitive to CSF-1 blockade [102]. However, one can surmise that, like Kupffer cells, the cells immediately adjacent to the macrophage are important niche members. In the case of LCMs, the mesothelium and subcapsular fibroblasts are likely important niche members. Also, diffusible factors from the peritoneal cavity may be necessary.

The niche for homeostatic liver bile duct and central vein macrophages is unknown. Bile duct-like LAMs have been shown to be derived from CX3CR1^+^/CCR2^+^ monocytes in disease models [130], and LAMs in other organs are monocyte derived [106], but whether that holds true for homeostatic bile duct macrophages remains to be definitively proven. Transcriptional analyses of these cells suggests a monocyte cell of origin for bile duct macrophages [25], but as of this writing, no conclusive lineage-tracing based approaches have proven the ontogeny of these cells. As with Kupffer cells, the neighboring cells, such as the mesothelium and fibroblasts in the capsule, and epithelial glands in the biliary system [131], are likely sources of maintenance signals for macrophages in these structures.

## Dynamics of liver macrophages during liver perturbations

As discussed above, liver resident macrophages, such as Kupffer cells, can either be relatively quiescent or rapidly turned over, such as subcapsular macrophages (Fig. 2). However, in the face of tissue disturbances, liver macrophages can be rapidly mobilized or replaced depending on the stimulus. The liver is prone to diverse insults, including acute injury, chronic injury, infection, and cancer metastases. These perturbations disturb or activate the homeostatic liver macrophage pool and may also cause the recruitment of additional macrophages into the liver. Here, we will highlight a few examples of the diverse macrophage response to select liver perturbations.

### Infection

While Kupffer cells can catch and clear many types of bacteria, specific pathogens can escape Kupffer cell-mediated killing. One example is *Listeria monocytogenes*, a foodborne bacteria that can cross the epithelial barrier and reach the bloodstream before being systemically disseminated. Kupffer cells that catch *Listeria* undergo necroptosis [132], which triggers the recruitment of circulating monocytes [133]. These monocyte-derived macrophages underwent proliferation within the liver, which depends on M-CSF and IL-4 [133]. Monocyte-derived macrophages can clear the infection from the liver, suggesting that their relatively immature state may present an advantage in clearing such infections. Indeed, monocytes are considered “more inflammatory” than adult Kupffer cells. *Salmonella enterica* is another example of a bloodborne pathogen that induces Kupffer cell death, monocyte recruitment, and eventual replacement of the Kupffer cell pool by monocyte-derived macrophages [133].

**Fig. 3:**
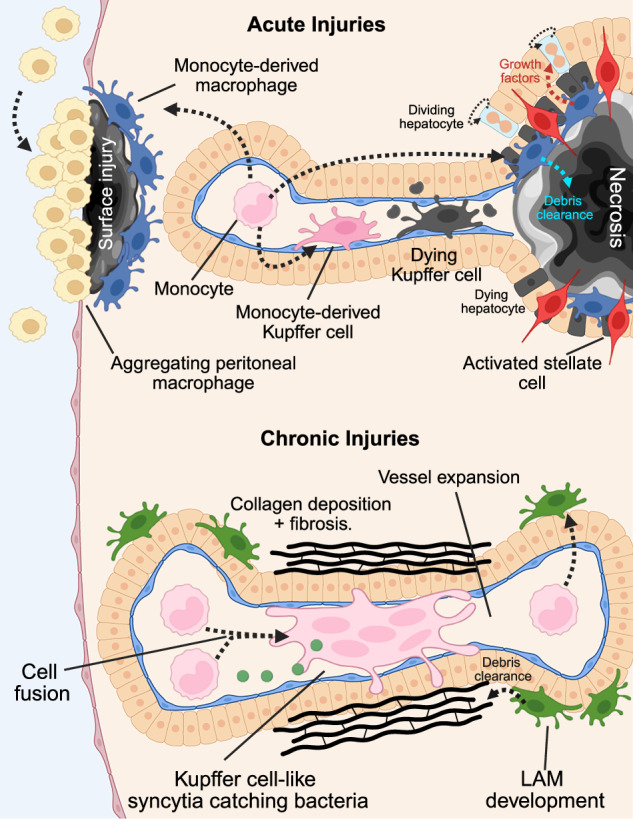
Dynamics of liver macrophages. In acute injuries (top) monocytes can replace most macrophage pools, including Kupffer cells. In necrotic injuries (top right), monocyte derived macrophages are recruited to the site of injury, and activate hepatic stellate cells, which provide a contractile force to close the lesion. Macrophages are also involved in debris clearance and provide signals for hepatocyte proliferation nearby, especially in partial hepatectomy (not shown). In sterile injuries to the liver surface, monocyte derived macrophages are recruited, and peritoneal macrophages aggregate on the surface. During chronic injuries (bottom), collagen and fibrosis accumulates, and vessels expand in diameter. Monocyte derived macrophages fuse into Kupffer cell-like syncytia, which catch bloodborne pathogens. Lipid-associated macrophages (LAMs) expand in number in many injuries and assist in debris clearance. Created in BioRender. Nusse, Y. (2025) https://BioRender.com/o6hji3p

In bacteria such as *Staph aureus*, which Kupffer cells can usually effectively clear, some bacteria can establish an intracellular niche within Kupffer cells. Colonies of Staph can begin replicating in phagolysosomes that do not produce a sufficient amount of ROS [68]. This is clinically important because reservoirs of intracellular Staph may escape treatment with antibiotics [134]. Additionally, the growth of intracellular Staph eventually overwhelms the Kupffer cells, causing them to lyse. Presumably, like other contexts where Kupffer cells are lost, monocytes can replace them after Staph induced lysis (Fig. 3).

### Tissue loss—partial hepatectomy

Perhaps the best example of the liver’s regenerative capacity is partial hepatectomy, in which up to two-thirds of the liver mass is surgically removed, followed by the dramatic regrowth of the organ [135]. This classic model has been used for decades to study organ regeneration and cell proliferation, and many studies have pointed to the role of macrophages in this phenomenon. Upon resection, the parenchymal cells in the liver begin proliferating, as well as undergoing hypertrophy [136]. This process depends on several classical developmental signaling factors but also requires signals associated with innate immune cells, such as the cytosolic nucleic acid sensors STING and MAVS [137]. Kupffer cells are thought to be key coordinators of this response through the secretion of TNFα, IL6, and TGFβ, which have been proposed to directly act on hepatic stellate cells or hepatocytes to trigger cell proliferation and expansion [138, 139]. When macrophages are depleted, using clodronate-loaded liposomes, liver regeneration is impaired [140]. However, it should be noted that administration of clodronate-loaded liposomes broadly depletes macrophages and has effects on monocytes and other cells [141], so the specific role of Kupffer cells in liver repair after partial hepatectomy should be more carefully examined using more specific depletion strategies such as *Clec4f-DTR* mice.

After partial hepatectomy, the Kupffer cell pool must be regenerated along with the rest of the parenchyma. In contrast with several other situations in which Kupffer cells are lost, during regeneration after partial hepatectomy, new Kupffer cells are not primarily derived from monocytes but rather through replication and apparent migration of the endogenous yolk-sac derived adult macrophages that remain [142]. While monocytes do contribute to new Kupffer cell pools, blocking monocyte recruitment actually enhanced local Kupffer cell proliferation, although ultimately monocyte impairment was deleterious for regrowth [143]. Kupffer cell replication was driven by IL-6, the source of which appears to be derived from both myeloid cells and hepatocytes [142]. Why, in this specific injury, the growth of the macrophage pool is driven by the proliferation of the endogenous Kupffer cells and not through the recruitment of monocytes is a fascinating question. One may speculate that this is due to the fact that new niches for the macrophage must be established in the regenerated organ, whereas most other models feature an open niche that can receive monocytes.

A recent paper examining liver regrowth after partial hepatectomy found that macrophages activated by glutamate signaling are critical for liver regeneration [144]. *Uri1*, a prefoldin-like chaperone expressed by hepatocytes near the central vein, converts glutamate into glutamine but is lost after partial hepatectomy, resulting in elevated glutamate levels. Genetic deletion of *Uri1* boosted glutamate levels and increased the speed of liver regeneration through increased hepatocyte proliferation. Furthermore, *Uri1* mutants had increased numbers of Csf-1R^+^ macrophages (which are likely a combination of Kupffer cells and monocyte-derived macrophages given their expression of *Ly6C* and *CCR2*), which, when reduced with a CSF-1R inhibitor, impaired liver regeneration. This phenomenon is thought to occur through glutamate signaling through HIF1a, which drove *Wnt3* expression in macrophages. *Wnt3* deletion in myeloid cells prevented proper liver regeneration, and Hif1a was required for *Wnt3* expression. This study suggests that after partial hepatectomy, rising glutamate levels rapidly recruit bone marrow-derived macrophages into the liver, which express *Wnt3* and trigger hepatocyte proliferation. Glutamate supplementation facilitated liver regeneration, raising the possibility that this could be a therapeutic target. This fascinating study illuminates how macrophages are recruited to the liver after partial hepatectomy and promote repair by secretion of key growth factors. The precise ontogeny of these macrophages is a fascinating area for further exploration.

### Acute injuries—sterile injury

Another model of liver damage that features the recruitment of macrophages is a sterile surgical burn injury, which ablates a small area on the surface of the liver (Fig. 3). This model triggers the recruitment of a diverse set of immune cells, including neutrophils [145, 146], iNKT cells [53], and at least two types of macrophages. Ly6C^+^ monocytes are rapidly recruited to the site of injury, where they switch from a CCR2^high^ CX3CR1^low^ phenotype to a CCR2^low^ CX3CR1^high^ state, which is dependent on IL-4 and IL-10 signaling [147]. This switch was necessary to clear debris from the injury site or resulted in delayed healing. In this model, it remains to be determined whether these monocytes fully differentiate into mature macrophages and whether they adopt a Kupffer-like state or become another type of macrophage.

Another source of liver macrophages that are uniquely specific to injury are peritoneal macrophages [148, 149]. These free-floating macrophages inhabit the body cavities, but upon injury to the visceral organs, they rapidly adhere and form aggregates akin to platelets [150–153] (Fig. 3). During acute liver injuries that damage the mesothelium, peritoneal macrophages attach to the surface of the liver and differentiate [154]. In some studies, this has led to enhanced repair whereas others failed to see any effect of these cells [155]. Similarly, cancerous metastases that disturb the liver mesothelium also attract peritoneal macrophages [156]. In these contexts, peritoneal macrophage recruitment to the liver is thought to promote tissue regrowth or tumor growth in cancer. However, whether these macrophages ultimately integrate into the homeostatic liver macrophage pool upon return to homeostasis remains unclear [155].

### Acute injuries—toxins and inflammatory injuries

Several laboratory models of acute liver injury attempt to mimic damage driven by toxins, such as alcohol poisoning, acetaminophen overdose, or acute inflammation. These include acute carbon tetrachloride, acetaminophen overdose, and T-cell activation, amongst several others. While the mechanisms of these models are different, they often result in hepatocyte death, development of necrotic nodules in the liver, recruitment of macrophages, and eventual repair [23]. A singular role for macrophages in these injuries is often nebulous, as differing studies point to macrophages being reparative or deleterious. Furthermore, most studies of liver macrophages do not precisely distinguish between endogenous yolk-sac-derived Kupffer cells, monocyte-derived Kupffer cells, or other macrophages due to technical limitations and a lack of markers for these various types of macrophages at the time.

In acetaminophen overdose, hepatic necrosis activates innate immune cells, results in the loss of the endogenous Kupffer cell population, and recruits monocyte-derived macrophages [157, 158] (Fig. 3). However, the function of macrophages in injury resolution is seemingly opposed. Depleting monocytes with an anti-CCR2 antibody (which spares endogenous Kupffer cells) exacerbates liver injury [157]. However, *CCR2* deletion, which impairs monocyte recruitment, may benefit healing [158]. Whether this reflects a lack of CCR2 monocytes from birth in knockout models versus antibody depletion in adults remains to be elucidated. The Kupffer cell pool is thought to recover via self-replication in this context. Still, these conclusions are based on observations of proliferating cells marked by negative expression of *CX3CR1* (and therefore assumed to be Kupffer cells) rather than definitive lineage tracing. Additionally, in acetaminophen damage, Kupffer cells secrete IL-1a in response to damage signals sensed by TLR signaling [159], which is thought to recruit inflammatory monocytes that further damage the liver. Again, in this context, F4/80^+^ CD11b^+^ cells were assumed to be Kupffer cells, so it is possible that other macrophages may be the source of IL-1a. Similarly, in response to toxic injury driven by carbon tetrachloride, or bile duct ligation (in which draining bile ducts are surgically ligated, resulting in injury to the cholangiocytes and fibrosis [160]), monocytes home to the liver and differentiate into Kupffer cells, which eventually outcompete endogenous Kupffer cells [161]. This is perhaps because monocyte-derived Kupffer cells are more proliferative and less prone to apoptosis based on single-cell RNAseq signatures.

In a model of acute inflammation, Concanavalin-A treatment activated T-Cells, which results in an inflammatory cascade releasing a number of pro-inflammatory cytokines, such as TNFa, IFNg, and others, resulting in necrotic lesions in the liver [162] (Fig. 3). Like other lesions, the liver can recover by mobilizing hepatocyte proliferation, which, along with the regrowth of other parenchymal cells, heal the injuries within a few days [163]. After Concanavalin-A injury, monocyte-derived macrophages are recruited to lesions [164]. These are likely not Kupffer cells, as they were *Clec4f* negative and were labeled via a GFP bone marrow transplantation experiment. Monocyte-derived macrophages formed a ring around the necrotic zone and secreted Notch ligands, signaling nearby hepatocytes to express *Sox9* and adopt a pro-survival, yet proliferatively silent state. This presumably limited cell death and contained the spread of necrosis. Furthermore, monocyte-derived macrophages closely interacted with activated hepatic stellate cells which had invaded the necrotic region. Activated hepatic stellate cells are highly contractile cells that provided a mechanical force to close the necrotic area. Depleting monocyte-derived macrophages with clodronate liposomes, or *CCR2* knockout, prevented the activation and invasion of hepatic stellate cells and impaired injury resolution. A single-cell RNAseq analysis revealed that two populations of monocyte-derived macrophages appear to be recruited at the necrosis site, one that expressed *C1q* and one that expressed *Pdgfb*. The *C1q*^+^ population seemed to have functions related to clearance of debris, based on expression of cathepsin B, legumain, and apolipoprotein E. Inhibition of cathepsin B, or C1q knockout impaired debris clearance and resulted in more extensive lesions, confirming the function of this subtype of monocyte-derived macrophage. Conversely, the *Pdgfb*^*+*^ population appears to be responsible for activating hepatic stellate cells, as *Pdgfb* is a known activator of stellate cells and myeloid-specific deletion of *Pdgfb*-impaired stellate cell activation and lesion closure. This study suggests that multiple subtypes of monocyte-derived macrophages cooperate with the adjacent liver parenchyma to limit damage caused by T-cell activation, clear debris, and close the lesion via mechanical forces exerted by activated hepatic stellate cells.

### Chronic injury

When the liver is exposed to long-term damage, such as in alcohol abuse, chronic viral infection, and other perturbations due to metabolic dysfunction, chronic damage can result in long-term inflammation, architectural changes to the liver lobules, and pathological scarring, known as cirrhosis [165, 166]. In the laboratory, one model used to study the liver in this context is through chronic administration of carbon tetrachloride, which damages the liver hepatocytes and results in liver fibrosis and eventual cirrhosis [167]. This results in a profound remodeling of the liver architecture, including the deposition of collagen and the development of abnormally enlarged and dysfunctional blood vessels [168, 169] (Fig. 3). These abnormal blood vessels exhibit rapid and turbulent blood flow, while in the sinusoids, blood flow is reduced due to vessel constriction [70]. This results in Kupffer cell dysfunction [170], manifested by an inability to catch and kill bacteria effectively, and a loss of the key Kupffer cell maturity markers, such as CRIg and TIM4. This is due to alterations in the Kupffer cell niche, notably loss of the interaction between Kupffer cells and hepatic stellate cells normally mediated via pseudopods extending across the Space of Disse. Loss of these pseudopods resulted in repression of the key Kupffer cell transcription factor LXRα and down regulation of bacterial catching molecules such as CRIg [70].

Intriguingly, fibrotic livers can still fulfill some filtration functions and clear bloodborne bacteria. This is accomplished by establishing Kupffer cell-like syncytia, which are large multi-nucleated clusters of monocyte-derived macrophages located in the abnormally large vessels of the fibrotic liver (Fig. 3). These clusters can rapidly catch bacteria and express CRIg, like Kupffer cells, but in fact are derived from monocytes. Furthermore, the formation of these macrophage syncytia was dependent on bacterial signals that leak through a permeable epithelial gut barrier and the cell adhesion molecule CD44 and scavenger receptor CD36 [70]. This dynamic mechanism illustrates how liver macrophages can compensate in the face of radical changes to their microenvironment to fulfill their critical functions in maintaining the sterility of the bloodstream.

### Hepatic lipid-associated macrophages (LAMs)

Recently, it has become apparent that a specific type of macrophage plays a significant role in multiple types of liver injury – hepatic lipid-associated macrophages (LAMs). As mentioned above, hepatic LAMs are present in the homeostatic liver, localized near the bile ducts, and express markers such as *CD9*, and *Trem2*. However, several recent studies have highlighted the critical role of these macrophages in diverse forms of liver injuries, suggesting that the recruitment of LAMs may be a conserved response to liver damage in both acute and chronic forms.

In Metabolic dysfunction-associated steatohepatitis (MASH), which is an advanced form of Metabolic dysfunction-associated steatotic liver disease (MASLD) associated with obesity, diabetes, and other metabolic disorders, the liver is damaged due to accumulation of fat and becomes chronically inflamed and fibrotic [171–173]. In the progression of this disease, Kupffer cells are lost and are replaced by monocyte-derived macrophages, which differentiate towards Kupffer cells [119, 174] (Fig. 3). However, other monocytes differentiate into a distinct subtype of macrophages that do not express Kupffer cell markers like CRIg, Clec4f, and Tim4 but instead adopt features of lipid-associated macrophages from adipose tissue, highlighted by expression of *Trem2*, *CD9*, and *Spp1/* Osteopontin [130, 175–177]. While these cells are expanded in models of MASLD, they also appear to be recruited in other liver injuries that are not thought to be primarily driven by fat accumulation in the liver or metabolic disorders. Hepatic LAM-like macrophages are observed in liver damage-driven acetaminophen overdose, derived from both monocytes, but also Kupffer cells [178]. LAM-like Kupffer cells express the Kupffer cell markers Clec4f, *Vsig4*/CRIg, and *Marco*, but also the LAM markers *Trem2*, and *CD9*, among others. Unlike Kupffer cells, after acetaminophen LAM-like Kupffer cells were located closer to the central vein (the region of liver damage), unlike the portal preference of normal Kupffer cells. Lineage tracing and bone marrow chimeras suggest that LAMs are derived from bone marrow-derived monocytes, while LAM-like Kupffer cells are not, suggesting these cells are the result of Kupffer cell plasticity. Mechanistically, it appears that the LAM state is driven by phagocytosis of apoptotic and necrotic cells [176–178]. *Trem2*, a conserved marker of LAMs, seems to be functionally important, as deletion of *Trem2* in macrophages (both Kupffer cell and monocyte-derived) impaired injury repair via lack of debris clearance and consequently increased fibrosis [130, 176]. Deletion of *Trem2* in either of these pools had no effect, suggesting that Kupffer cell-derived and monocyte-derived LAMs are functionally redundant [178].

LAMs and LAM-like Kupffer cells were also observed in carbon tetrachloride-driven damage [178]. LAM-like macrophages are also observed in T-Cell mediated liver damage (where they appear related to the *C1q*+ debris clearing monocyte-derived macrophages) [164], but whether Kupffer cells can adopt a LAM phenotype in this model has not been definitively explored.

### Human macrophages and relevance for disease

Within this review we have mostly focused on data gleaned from mouse models. However valuable studies have been conducted on liver samples from human patients as well as in vitro studies which have provided additional insight and confirmation of biological interpretations made from animals. In MASH, human TIMD4^+^ MARCO^+^ Kupffer cells are lost and are replaced by monocytes, which display a pro-inflammatory phenotype [179]. Single cell transcriptional profiling has revealed that in primary sclerosing cholangitis, in which the bile ducts are damaged and liver scarring occurs, resident macrophages are reduced and monocyte derived macrophages display an “exhausted” response to immune stimulus [180]. Additionally, similar studies examining liver cirrhosis have uncovered evidence of Kupffer cell loss and *TREM2*^+^*CD9*^+^*SPP1*^+^ LAM expansion, which are derived from monocytes based on transcriptional trajectories and in vitro studies [181, 182]. Cirrhotic LAMs may promote fibrosis through stellate cell activation through TNF and PDGF signaling, as shown in culture. These studies largely point to a loss of the homeostatic Kupffer cell population, disruption of the Kupffer cell niche, and expansion of monocyte derived macrophages with divergent functions as a conserved feature in human liver disease.

## Liver macrophages in the future

There has never been a better time to study liver macrophages, facilitated by the development of exciting new technologies, tools, and ideas. The advent of single-cell transcriptomics, spatial transcriptomics, spectral flow cytometry, multiplex immune-fluorescence, and single-molecule in situ hybridization has allowed unprecedented insight into the populations of liver macrophages that, until now, could only be characterized by a few simple markers. This has allowed researchers to identify entirely new types of macrophages in the homeostatic liver, important intermediary states during differentiation and maturation, and shed light on the complex dynamics at play during liver inflammation.

Beyond these technologies that facilitate the identification and description of macrophages, new genetic tools have enabled us to functionally test the role and behavior of liver macrophages with unprecedented precision. While in previous years, the most reliable method to deplete liver macrophages was to broadly deplete them with toxic drugs that have unwanted off-target effects, mutants such as *CCR2* knockouts and *Clec4f-Cre DTR* mice allow researchers to specifically deplete targeted populations while sparing other liver macrophages, also with temporal control. Depleting antibodies and pharmacological tools complement this approach. Tracing tools such as the *Ms4a3-Cre*, *Clec4f-Cre*, and *CX3CR1-CreEr* lines allow researchers to carefully dissect the lineage of macrophages like never before. Other genetic tools including photo-activatable-fluorescent reporters and in vivo methods to detect cell interactions used on advanced super-resolution microscopy platforms allow intra-vital imaging experiments that reveal macrophages’ real-time behavior in living tissue.

All these newly developed tools should facilitate our understanding of liver macrophage biology in the coming years and hopefully resolve confusion about the role of macrophages in liver disease, especially regarding their regenerative or deleterious contribution to repair. Uncovering the role of these critical cells in liver diseases has never been more within reach, which should make real-world impacts on human patients suffering from liver pathologies.
